# Effect of Different Disinfectants on Staphylococcus aureus and Candida albicans Transferred to Alginate and Polyvinylsiloxane Impression Materials

**DOI:** 10.5681/joddd.2009.030

**Published:** 2009-12-15

**Authors:** Fereydun Parnia, Ali Hafezeqoran, Elnaz Moslehifard, Farhang Mahboub, Mohammadreza Nahaei, Mohammad Akbari Dibavar

**Affiliations:** ^1^ Assistant Professor, Department of Prosthodontics, Faculty of Dentistry, Tabriz University of Medical Sciences, Tabriz, Iran; ^2^ Professor, Department of Microbiology, Faculty of Medicine, Tabriz University of Medical Sciences, Tabriz, Iran; ^3^ MSc, Department of Microbiology, Faculty of Medicine, Tabriz University of Medical Sciences, Tabriz, Iran

**Keywords:** Alginate, Candida albicans, disinfection, impression materials, silicone elastomers, Staphylococcus aureus

## Abstract

**Background and aims:**

Several products have been marketed for disinfecting impression materials. The present study evaluated the effect of Deconex, Micro 10, Alprocid and Unisepta Plus sprays on *Staphylococcus aureus* and *Candida albicans* transferred to alginate and polyvinylsiloxane impression materials.

**Materials and methods:**

A total of 180 impressions of a maxillary model (90 alginate and 90 polyvinylsiloxane im-pressions) were taken for the purpose of this in vitro study. Half of the impressions were infected with Staphylococcus au-reus and the other half were infected with *Candida albicans*. Then the microorganisms were cultured and their counts were determined. Subsequently, the impressions were divided into groups of 15 impressions each. Each group was disinfected with Deconex, Micro10, Alprocid and Unisepta Plus according to manufacturers' instructions except for the control group. The culturing procedure was repeated after disinfection and microbial counts were determined again. Data was analyzed by ANOVA and paired-sample t-test.

**Results:**

There were statistically significant differences in the means of *S. aureus* and *C. albicans* counts before and after the use of disinfectants (P < 0.05). The use of the four disinfectants reduced *S. aureus* counts to zero in 80% of the cases. There were no statistically significant differences in *S. aureus* count reductions between the four disinfectants evaluated (P = 0.31). Micro 10 was more effective on alginate; Deconex was more efficient for polyvinylsiloxane and Alprocid had a better efficacy in both impression materials in eliminating *C. albicans* (P < 0.05).

**Conclusion:**

All the disinfectants evaluated have high disinfecting postentials.

## Introduction

Alarge number of microorganisms live and grow in the oral cavity, which might be transmitted to others.^1 - 3^ Dental procedures transfer the microorganisms in the saliva and blood to surfaces, impressions and dental instruments, contaminating them.^4^ Infection control consists of not only preventing the transmission of infectious diseases such as AIDS, hepatitis and tuberculosis but also preventing cross-contamination of pathogenic oral microflora.^2 - 6^ Therefore, infection control is an important responsibility of dental practitioners.

In some countries protocols concerning the disinfection of materials sent to dental laboratories have been in effect for some years; however, such measures are not observed in all the countries.^7^ Since impression materials and occlusal record indices cannot be heat-sterilized, chemical disinfectants are the choice procedures for disinfecting such materials.^8^ At present, a wide range of disinfectants are available, which are classified into weak, moderate and strong in different texts based on their disinfecting potentials and capabilities.^9^ New products and disinfecting agents are continually being marketed with different brands and capabilities; however, their real efficacies are not known in comparison to other commonly-used agents. Dental practitioners always try to use very effective agents with the least side effects;^10^ therefore, it is necessary to compare the disinfecting potential of various products for proper infection control.

The present study evaluated the disinfecting potentials of Deconex, Micro10, Alprocid and Unisepta Plus sprays on *S. aureas* and *C. albicans* transferred to alginate and polyvinylsiloxane impression materials.

## Materials and methods

A model of maxilla was used to take 180 impressions for the purpose of this experimental study. A total of 90 impressions were made with alginate (Tropicalgin, Zhermark, Italy) and 90 other impressions were made with polyvinylsiloxane (Examix, NDS, GC America, Illinois, USA). Metallic trays which had been in an autoclave at 121˚C for 15 min were used. The trays were kept in sterile nylon bags until they were used. The impression materials were used according to manufacturers' instructions. Principles of asepsis were strictly followed and observed during impression taking, including the use of face masks and sterile gloves. Alginate was mixed with distilled water and all the impressions were taken by one person. A swab was used to contaminate the impressions with *S. aureus* (*S. aureus* ATCC 29213) and *C. albicans* (isolated from acute lymphoblastic leukemia patients undergoing chemotherapy). McFarland's 0.5 standard concentration was used to prepare the microbial suspension. An isolated monocolony of the microorganisms under study was used to ensure microorganism purity. Special culture media (mannitol salt agar for *S. aureus*, and Sabouraud's dextrose agar for *C. albicans*) were used for the purpose of the study. The controls consisted of 15 alginate and 15 polyvinylsiloxane impressions contaminated with *S. aureus*, and 15 alginate and 15 polyvinylsiloxane impressions contaminated with *C. albicans*, in which no disinfecting agents were used. All the samples underwent a culturing procedure and microbial counts were determined. Each group (15 alginate and 15 polyvinylsiloxane impressions each) was disinfected by spraying one of the four disinfecting agents for 30 seconds, according to manufacturers' instructions: Deconex spray (Deconex Solarsept, Borer Chemie AG, Switzerland); Micro 10 spray (Micro 10 Unident, Geneve, Switzerland); Alprocid spray (Alprocid, Moosweisenstr, Schwarzwald, Germany); Unisepta Plus spray (Unisepta Plus, Unident, Geneve, Switzerland). The culturing procedure was repeated and microbial counts were determined for all the samples.

The disks from each impression were placed in 10 mL of phosphate-buffered saline solution (PBS) for 40 seconds to make sure that all the microorganisms attached to the impression material were detached. Subsequently, the solution was transferred to a sterile test tube; 10 mL of the solution was transferred to the blood agar culture media and incubated for 24 h. Gram staining was carried out and bacterial CFUs were calculated by the formula CFU/mm ^2^ of impression surface = (CFU/mL × ds/pr ^2 ^, in which “d” represents dilution coefficient, “r” is the radius of the metallic cylinder and “CFU” is colony-forming units.

SPSS 13 was used for statistical analysis. Paired-sample t-test was used to compare microbial counts before and after the disinfection procedures; ANOVA was used to compare bacterial counts between the four impression materials.

## Results

**Figure 1:**
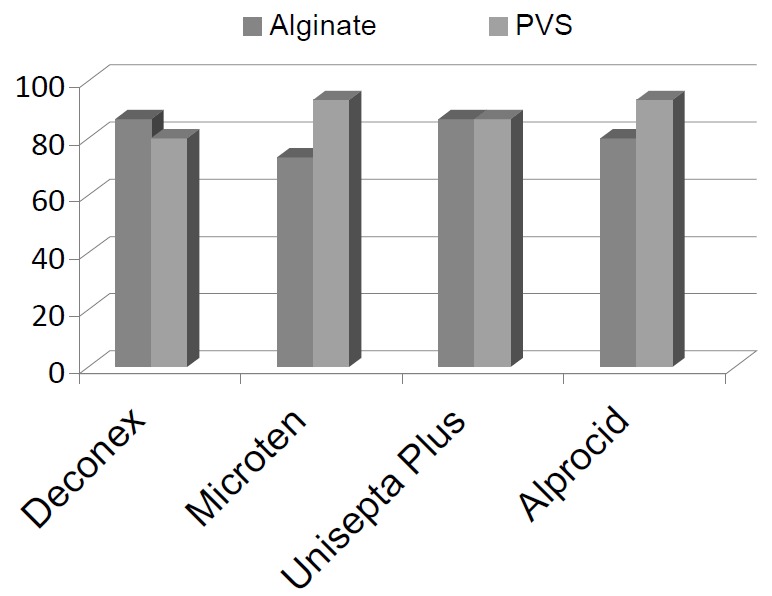
Percentage of impressions devoid of *S. aureus* subsequent to the use of disinfecting agents, separately for both impression materials.

The means of *S. aureus* counts in alginate impressions before and after disinfection with Deconex were 2644.76 ± 2383.84 and 4.22 ± 1.50, respectively; the counts were 1373.27 ± 1103.52 and 6.23 ± 3.01 for Micro 10 ; 1163.19 ± 707.04; and 2.64 ± 1.00 for Unisepta Plus; and 598.16 ± 354.46 and 2.59 ± 1.15 for Alprocid, respectively. The differences between bacterial counts before and after disinfection with disinfectants were statistically significant (P < 0.05). The results demonstrated that Deconex reduced bacterial counts to zero in 86.7% of cases. Micro 10, Unisepta Plus and Alprocid reduced *S. aureus* counts to zero in 73.3%, 86.7% and 80% of cases, respectively, in alginate impressions (Figure 1).

In polyvinylsiloxane impressions *S. aureus* counts before and after disinfection with Deconex, Micro 10, Unisepta Plus and Alprocid were 941.71 ± 705.70 and 6.15 ± 2.51, 460.30 ± 233.19 and 1.94 ± 0.50, 1968.40 ± 1187.01 and 2.64 ± 1.00, and 599.72 ± 586.22 and 1.94 ± 0.50, respectively. In all those cases there were statistically significant differences between bacterial counts before and after the use of disinfectants (P < 0.05). In polyvinylsiloxane impressions, Deconex, Micro10, Unisepta Plus and Alprocid completely eliminated the bacteria in 80%, 93.3%, 86.7% and 93.3% of cases, respectively (Figure 1).

**Figure 2:**
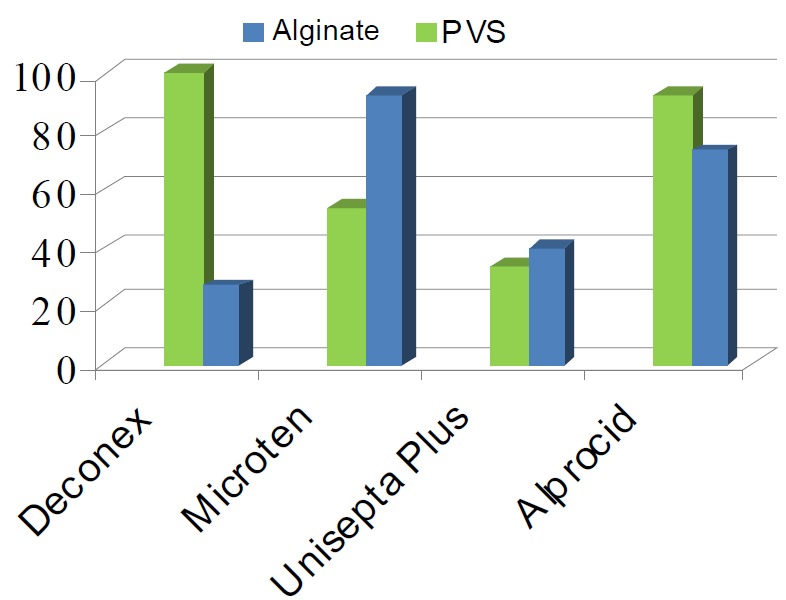
Percentage of impressions devoid of *C. albicans* subsequent to the use of disinfecting agents, separately for both impression materials.

*C. albicans* counts in alginate impressions before and after the use of Deconex, Micro 10, Unisepta Plus and Alprocid were 571.17 ± 292.92 and 53.48 ± 35.67, 117.08 ± 68.95 and 1.94 ± 0.50, 644.24 ± 312.01 and 9.38 ± 6.52, 203.97 ± 134.63 and 3.44 ± 2.00, respectively. The differences in the counts before and after the use of disinfectants were statistically significant (P < 0.05). The results indicated that in alginate impressions Deconex, Micro 10, Unisepta Plus and Alprocid completely eliminated *C. albicans* in 26.7%, 93.3%, 40% and 73.3% of cases, respectively (Figure 2).

In polyvinylsiloxane impressions *C. albicans* counts before and after the use of Deconex, Micro 10, Unisepta Plus and Alprocid were 252.26 ± 92.00 and 0.00 ± 0.00, 348.74 ± 221.68 and 9.64 ± 5.52, 2138.43 ± 121.51 and 24.78 ± 20.09, and 292.96 ± 158.62 and 2.64 ± 1.00, respectively. In those cases, the differences in the counts before and after the use of disinfectants were also statistically significant (P < 0.05). In polyvinylsiloxane impressions Deconex, Micro 10, Unisepta Plus and Alprocid completely eliminated Candida species in 100%, 53.3%, 33.3% and 86.7% of cases, respectively (Figure 2). ANOVA indicated a statistically significant difference among the study groups (P < 0.05). The results of the present study demonstrated that Micro 10 was more effective in alginate impressions, Deconex was more effective in polyvinylsiloxane impressions and Alprocid was more effective in both impression materials in eliminating *C. albicans* compared to other disinfecting agents evaluated (P < 0.05).

## Discussion

Micro 10, Deconex, Unisepta Plus and Alprocid are disinfecting agents introduced in the 1990s,^11^ which are widely used in Iran by dental practitioners. A large number of studies have evaluated disinfecting properties of Micro 10 and Deconex; however, only a few studies have evaluated Alprocid and Unisepta Plus.

In the present study, Deconex completely eliminated *S. aureas* from 88.7% of alginate impressions and 80% of polyvinylsiloxane impressions and considerably reduced bacterial counts in the remaining cases. In addition, Deconex eliminated *C. albicans* from alginate impressions in 26.7% of cases and reduced their counts from 571.17 to 35.67 in the remaining cases. Cheristensen^12^ classified Deconex as a moderately potent disinfecting agent since it was effective on all bacterial and fungal strains in their study, which is consistent with the results of the present study. In a study carried out by Ghahramanloo et al^13^ Deconex ranked third after Chloro-Sol (0.525% sodium hypochlorite) and Sanosil Super 25. In that study Deconex removed gram-positive and gram-negative bacteria in 70.8% of cases. According to the results of the present study, Micro 10 eliminated *S. aureus* in 73.3% of aligrate impressions and in 93.3% of polyvinylsiloxane impressions. In addition, Micro 10 eliminated *C. albicans* in 93.3% of alginate and 33.3% of polyvinylsiloxane impressions. Javaheri and Zanganeh^1^ evaluated the disinfecting properties of Micro 10 and Deconex at three intervals and reported that these two disinfectants were effective on different bacteria at the three intervals evaluated.

Unisepta Plus and Alprocid are effective on *S. aureus* in a similar manner to Micro 10 and Deconex. Therefore, they can be used as proper disinfectants. The four disinfectants evaluated were not similarly potent in eliminating *C. albicans*. Micro 10 was more effective on alginate impressions; Deconex was more effective on polyvinylsiloxane impressions; and Alprocid was more effective on both impression materials compared to other disinfectants. It is suggested that the effect of the duration of spraying and distance of spraying the impression materials be evaluated in future studies.

## Conclusion

The results of the present study demonstrated that all the four disinfectants evaluated have a similar and identical effect on *S. aureus* in both impression materials. These four disinfecting agents showed that they are highly potent and eliminate more than 80% of bacteria. The effect of all the agents on *C. albicans* is weak except for the effect of Micro 10 on alginate, Deconex on polyvinylsiloxane and Alprocid on both impression materials.
